# Statement of Retraction: CircRNA hsa_circ_0070659 predicts poor prognosis and promotes non-small cell lung cancer (NSCLC) progression via microRNA-377 (miR-377) / Ras-Associated Binding Protein 3C (RAB3C) pathway

**DOI:** 10.1080/21655979.2026.2667632

**Published:** 2026-05-21

We, the Editors and Publisher of the journal *Bioengineered*, have retracted the following article from publication.

Meng, Q., Li, Y., Sun, Z., & Yang, X. (2022). CircRNA hsa_circ_0070659 predicts poor prognosis and promotes non-small cell lung cancer (NSCLC) progression via microRNA-377 (miR-377) / Ras-Associated Binding Protein 3C (RAB3C) pathway. *Bioengineered*, *13*(6), 14578–14594. https://doi.org/10.1080/21655979.2022.2091572  

Since publication, significant concerns have been raised by a third party about the integrity of the data and reported results in the article.  

When approached for an explanation, the authors have provided their original data and answered our queries, but these do not sufficiently address the concerns.   

As verifying the validity of published work is core to the integrity of the scholarly record, we are therefore retracting the article. The authors have been informed of this decision. 

We have been informed in our decision-making by our policy on publishing ethics and integrity and COPE guidelines.   

The retracted article will remain online to maintain the scholarly record and will be digitally watermarked on each page as ‘Retracted’.  

